# A Pilot Study for the Characterization of *Bacillus* spp. and Analysis of Possible *B. thuringiensis*/*Strongyloides stercoralis* Correlation

**DOI:** 10.3390/microorganisms12081603

**Published:** 2024-08-06

**Authors:** Elena Pomari, Pierantonio Orza, Milena Bernardi, Fabio Fracchetti, Ilenia Campedelli, Patrick De Marta, Alessandra Recchia, Paola Paradies, Dora Buonfrate

**Affiliations:** 1 Department of Infectious, Tropical Diseases and Microbiology, IRCCS Sacro Cuore Don Calabria Hospital, Negrar di Valpolicella, 37024 Verona, Italy; pierantonio.orza@sacrocuore.it (P.O.); milena.bernardi@sacrocuore.it (M.B.); dora.buonfrate@sacrocuore.it (D.B.); 2 Microbion srl, San Giovanni Lupatoto, 37057 Verona, Italy; f.fracchetti@microbion.it (F.F.); i.campedelli@microbion.it (I.C.); info@microbion.it (P.D.M.); 3 Department of Precision and Regenerative Medicine and Ionian Area (DiMePRe-J), Veterinary Section, University of Bari “Aldo Moro”, Valenzano, 70010 Bari, Italy; alessandra.recchia1@uniba.it (A.R.); paola.paradies@uniba.it (P.P.)

**Keywords:** *Strongyloides stercoralis*, *Bacillus thuringiensis*, cry5, DNA, microbiota, dog

## Abstract

Differentiating between *Bacillus* species is relevant in human medicine. *Bacillus thuringiensis* toxins might be effective against *Strongyloides stercoralis*, a nematode causing relevant human morbidity. Our first objective was to evaluate genomic and MALDI-TOF identification methods for *B. thuringiensis*. Our secondary objective was to evaluate a possible negative selection pressure of *B. thuringiensis* against *S. stercoralis*. PCR and Sanger were compared to MALDI-TOF on a collection of 44 *B. cereus* group strains. *B. thuringiensis* toxin genes were searched on 17 stool samples from *S. stercoralis*-infected and uninfected dogs. Metagenomic *16S* rRNA was used for microbiome composition. The inter-rate agreement between PCR, Sanger, and MALDI-TOF was 0.631 k (*p*-value = 6.4 × 10^−10^). *B. thuringiensis* toxins were not found in dogs’ stool. *Bacteroidota* and *Bacillota* were the major phyla in the dogs’ microbiome (both represented >20% of the total bacterial community). *Prevotella* was underrepresented in all *Strongyloides*-positive dogs. However, the general composition of bacterial communities was not significantly linked with *S. stercoralis* infection. The genomic methods allowed accurate differentiation between *B. thuringiensis* and *B. cereus*. There was no association between *B. thuringiensis* and *S. stercoralis* infection, but further studies are needed to confirm this finding. We provide the first descriptive results about bacterial fecal composition in dogs with *S. stercoralis* infection.

## 1. Introduction

*Bacillus* species are spore-forming, Gram-positive bacteria that include *Bacillus thuringiensis*, *B. cereus*, *B. cytotoxicus*, *B. anthracis*, *B. pseudomycoides*, *B. weihenstephanensis*, *B. toyonensis*, and *B. mycoidesare*. Being highly similar in genotype and phenotype, these bacteria are classified as part of the *B. cereus* group in taxonomy [1,2]. Unambiguous differentiation between *Bacillus* species can be relevant for human and animal health, but this is not usually possible with routine diagnostics [3]. For instance, *B. thuringiensis* is used as a biocontrol agent in agriculture, permitting a reduction in the amount of chemical products [4]. This is possible through the production of various crystal protein toxins (Cry5, Cry6, Cry12, Cry13, Cry14, Cry21, and Cry55) that exhibit a substantial biological activity against various insects, nematodes, and other pathogenic pests [5,6,7,8]. Previous studies reported *B. thuringiensis*-derived crystal protein Cry5B to be effective against a broad range of gastrointestinal parasitic hookworms [9] and *Strongyloides stercoralis* [10]. In particular, Cry5B was tested in vitro and in vivo on hamsters and dogs, demonstrating good efficacy against *Ancylostoma ceylanicum*, *Ancylostoma caninum*, and *Necator americanus* [9]. Regarding *S. stercoralis*, multiple stages, including the first larval stage (L1s), infective stage (iL3s), free-living adult stage, and parasitic female stage, were all susceptible to Cry5B, as indicated by the impairment of motility and decreased viability in vitro [10]. *Strongyloides stercoralis* is a soil-transmitted helminth that affects around 600 million people globally, causes a neglected tropical disease [11,12,13], and occurs in humans, non-human primates, dogs, cats, and wild canids [11]. Its zoonotic potential is under study [14,15,16]. Indeed, some authors have considered strongyloidiasis a zoonotic disease, while others have argued that the different hosts carry host specialized populations of *S. stercoralis*. In particular, clinical manifestations of *S. stercoralis* in humans and dogs range from asymptomatic to severe infection, with symptoms and signs involving mostly the intestines, respiratory tract, and skin [17]. Moreover, a potentially fatal syndrome (hyperinfection/dissemination) can develop; this is associated with immunosuppression in humans, while it can occur also in immunocompetent dogs [17]. The implementation of public health strategies for the control of *S. stercoralis* in endemic areas has been recommended by the WHO [18]. For instance, the strategies can include regular parasitological examinations of dogs and inspections of park soil, kennels, and dog shelters. Moreover, the strategies primarily entail mass drug administration of ivermectin, but caution should be paid to the possible emergence of drug resistance, considering the already-existing resistance to this drug observed in veterinary medicine [19,20]. Hence, additional strategies that could help to contain environmental contamination with *S. stercoralis* larvae might be useful for integration with mass drug administration. The primary objective of this study was to assess a MALDI-TOF and genomic lab pipeline for the identification of *B. thuringiensis* and its genes involved in the synthesis of the Cry toxins, such as cry5Ab, cry5Ac, and cry5Ba genes [21]. Our secondary objective was to investigate a hypothetical negative selective pressure of these toxins against *S. stercoralis* and a possible different bacterial composition in the gut microbiota of *S. stercoralis*-infected and uninfected dogs.

## 2. Materials and Methods

### 2.1. Study Setting and Population

A total of 44 bacterial strains (Table S1) from our collection were used for the genotyping and MALDI-TOF analyses. In particular, among this collection, 3 strains were used as positive controls for *B. thuringiensis* (NRRL B-18400, NRRL B-18765, and DSM 2046T) and 6 for *B. cereus* (LMG 6923T, LMG 12334, LMG 12335, LMG 17615, NCTC 11143, and ATCC 11778). Moreover, in order to explore the potential effect of *B. thuringiensis* on *S. stercoralis*, we analyzed 17 dog stool specimens (Table S2), a cohort enrolled in our previous study investigating the epidemiology of strongyloidiasis in Southern Italy [22]. The dogs’ samples were collected from a kennel and three farms located in Apulia Region and all samples were tested for *S. stercoralis* infection using both Baermann method and Real-Time PCR (rtPCR), as previously described [22]. In particular, three dogs resulted to be infected by *S. stercoralis* using both methods and another one tested positive only by rtPCR (see Table S2).

### 2.2. Mass Spectrometry

The *B. cereus* group strains were cultured on Columbia Agar with Sheep Blood (PB5039A Thermofisher, Monza, Italy) and incubated at 37 °C for 16/24 h. The mass spectrometry identification was performed by MALDI-TOF using the instrument Maldi Biotyper MBT smart (Bruker, Milan, Italy) with the MBT IVD Library DB 8326 March 2019 J and the software MBT Compass IVD v 4.1.100 (Bruker, Milan, Italy) following the manufacturer’s instructions.

### 2.3. DNA Extraction/Purification

For the *B. cereus* group strains, the total genomic DNA was extracted and purified from a 2 mL overnight culture using the Wizard Genomic DNA purification kit (Promega Corporation, Madison, WI, USA), following the manufacturer’s instructions. For the stool samples, the DNA was isolated from 200 mg using a Qiamp Fast DNA stool mini kit (Qiagen, Milan, Italy), according to the manufacturer’s instructions. The samples were eluted in 30 μL of elution buffer. The quality and quantity of DNA was analyzed using a NanoDrop One/Onec Spectrophotometer (Thermofisher, Monza, Italy) and a Qubit 4 Fluorometer (Thermofisher, Monza, Italy). The isolated DNA was stored at −80 °C until PCR and sequencing.

### 2.4. Genotyping of B. cereus Group Strains

The *B. cereus* group strains (*n* = 44) were first characterized by *Bacillus* species-specific PCR for a fragment of gyrase B gene (*gyrB*), followed by Sanger sequencing (Eurofins Genomics, Ebersberg, Germany) as previously described [23]. Moreover, the *B. cereus* group strains (*n* = 44) and the dog stools (*n* = 17) were analyzed using specific PCRs for the gene *ces* [24] coding *B. cereus* emetic toxin cereulide and for the genes *cry5Ab*, *cry5Ac,* and *cry5Ba* [21] coding for *B. thuringiensis* Cry toxins. In this analysis, we included the positive controls *B. thuringiensis* NRRL B-18400 and NRRL B-18765 for *cry* genes and *B. cereus* LMG 12,334 for *ces*. For all PCR experiments, the amplification products were loaded on a 1.5% agarose gel and visualized by exposure to ultraviolet (UV) light.

The Sanger sequences of the *gyrB* gene were aligned with Clustal X software v2.0 [25], obtaining a final consensus length of 645 nucleotides for the 44 strains under analysis and the type strain of the species *B. mycoides* DSM 2048T and *B. cytotoxicus* NVH 391-98T, for which the sequence was retrieved from the NCBI database. The consensus-sequence alignment was imported on MEGA software version 11 [26], and the Neighbor-Joining algorithm was used for the reconstruction of the phylogenetic tree. The evolutionary distances were computed using the Tamura–Nei model with complete deletion. The *gyrB* sequences of the strain *B. subtilis* subsp. *subtilis* BCRC 10255T was retrieved from the NCBI database and used as the outgroup.

### 2.5. Sequencing and Bioinformatic Analysis

To reduce the possible bias of biological/dietary/environmental interferents, we chose to analyze samples collected from dogs belonging to the same kennel, with sampling performed on the same day. For this purpose, among the total dogs cohort (*n* = 17), the metagenomics analysis was conducted on ten stool samples (three positives and seven negatives for *S. stercoralis*) collected from dogs living in the same kennel (Table S2). Libraries were prepared following the *16S* Metagenomic Sequencing Library Preparation protocol (Illumina, San Diego, CA, USA) in two amplification steps: an initial 35 cycle PCR amplification using 16S rDNA V3–V4-specific PCR primers (16S-341F 5′-CCTACGGGNBGCASCAG-3′ and 16S-805R 5′-GACTACNVGGGTATCTAATCC-3′) and a subsequent amplification that integrates relevant flow-cell binding domains and unique indices (NexteraXT Index Kit, FC-131-1001/FC-131-1002). Libraries were sequenced on a NovaSeq instrument (Illumina, San Diego, CA, USA) using 300 bp paired-end mode. Base calling, demultiplexing, and adapter masking were carried out through the Illumina BCL Convert v3.9.3 (https://emea.support.illumina.com/ (accessed on 18 January 2023)). The FASTQ sequences obtained were analyzed firstly using Kraken 2, which examines the k-mers obtained from a sequencing read sample with those produced from the Silva ribosomal RNA Database (release 138.1) available for Kraken 2 [27]. The taxonomic abundance for each taxon was estimated through Bracken [28]. In addition, the reads generated for the ten stool samples were analyzed through DADA2 version 1.8 [29] by the R 3.5.1 environment. DADA2 was run as described in https://benjjneb.github.io/dada2/tutorial.html (accessed on 20 July 2023), applying the following parameters: trimLeft equal to 30 and the truncLen option set to 270 and 200 for the forward and reverse fastq files, respectively. Taxonomic assignment was performed comparing the amplicon sequence variants (ASVs) predicted from DADA2 against the Silva ribosomal RNA Database (release 138.1) using the function assignTaxonomy and addSpecies.

### 2.6. Statistics

*B. cereus* and *B. thuringiensis* cases were summarized for each technique using frequencies and percentages. The agreement between MALDI-TOF, PCR, and Sanger was evaluated using Fleiss’ kappa coefficient.

## 3. Results

### 3.1. Identification Analysis of B. cereus Group Strains

**Figure 1:**
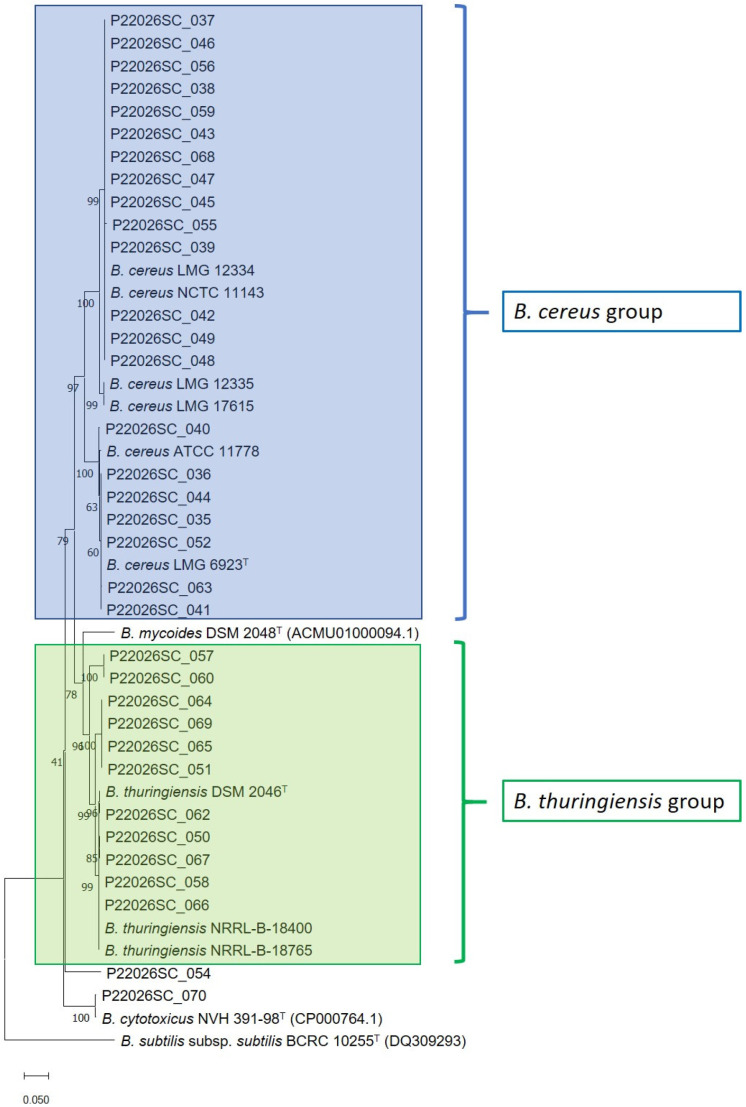
Neighbor-Joining phylogenetic tree based on the *gyrB* gene sequence comparison for the *Bacillus* strains under analysis with the corresponding sequence retrieved for the type strain *B. mycoides* DSM 2048^T^, *B. cytotoxicus* NVH 391-98^T^, and *B. subtilis* subsp. *subtilis* BCRC 10255^T^. The tree was reconstructed through MEGA11 with the Tamura–Nei model and complete deletion treatment for gaps. The accession numbers of the sequence deposited and/or available in NCBI database were reported in brackets.

MALDI-TOF and the specific *gyrB* PCR permitted us to identify *B. cereus*, *B. thuringiensis*, *B. mycoides*, *B. cytotoxicus,* and *B. subtilis* in the 44 *B. cereus* strains, as reported in Table S1. The results of Sanger sequencing are shown in Table S1 and Figure 1. Of note, one of the positive controls, *B. thuringiensis* NRRL B-18765, was misidentified as *B. cereus* by MALDI-TOF (Table S1). We then compared the data obtained from MALDI-TOF and the specific PCR followed by Sanger only on the *B. cereus* and *B. thuringiensis* results (*n* = 32 strains), excluding positive controls and other *Bacillus* species of the collection. We found 0.631 k (*p*-value = 6.4 × 10^−10^) inter-rate agreement between the three methods. As summarized in Table 1, all three methods identified *B. cereus* in twenty-one strains and *B. thuringiensis* in four strains, whilst seven strains resulted in *B. thuringiensis* by genomic approaches and were misidentified as *B. cereus* by MALDI-TOF.

### 3.2. Characterization for cry5Ab, cry5Ac, and cry5Ba by PCR

For a better differentiation, the Bacillus strains were investigated by specific PCRs for the genes *cry5Ab*, *cry5Ac* and *cry5Ba*, which code for the *B. thuringiensis* toxin Cry5B and for the gene *ces* coding for the *B. cereus* emetic toxin cereulide (Table S1). The analysis was performed using control strains *B. thuringiensis* NRRL B18765 and NRRL B-18400 and *B. cereus* LMG 12334, confirming the presence of *cry5Ab*, *cry5Ac,* and *cry5Ba* only in *B. thuringiensis* and the presence of *ces* only in *B. cereus,* as expected (Table S1). Apart from the controls described above, no other strains from the collection showed signals for all the analyzed genes (Table S1). For the study’s secondary objective, we tested the dogs’ stool for the genes *cry5Ab*, *cry5Ac,* and *cry5Ba*, finding no amplification in all samples (Table S3).

### 3.3. 16S Illumina

**Figure 2:**
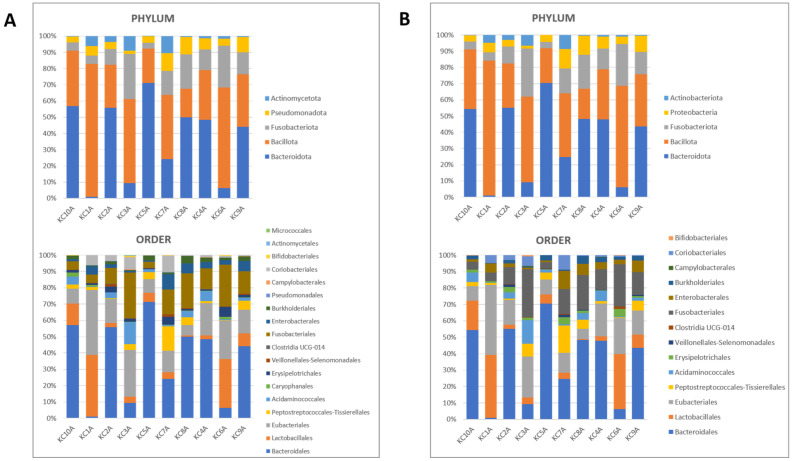
Phylum- and order-level gut microbiota composition in the fecal samples of 10 dogs. (**A**) Data obtained with Kraken2/Bracken. (**B**) Data obtained with DADA2.

Both Kraken2|Bracken and DADA2 identified *Bacteroidota* and *Bacillota* as the most abundant phyla (>20% as maximum value, Figure 2, Tables S5 and S6). In particular, four dogs in the present study were characterized by a reduction in *Bacteroidetes*, with a consequently increase in the ratio between *Bacillota* and *Bacteroidota*, presented by KC1A (dogs with frequent episodes of diarrhea), KC3A (dog manifesting some episodes of diarrhea), KC7A (dog positive for *S. stercoralis* infection in 2018), and KC6A (dog positive for *S. stercoralis* infection). The main orders were *Bacteroidales*, *Lactobacillales,* and *Eubacteriales* (>20% as maximum value, Figure 2, Tables S7 and S8). The main genera were *Prevotella*, *Streptococcus*, *Alloprevotella*, *Fusobacterium,* and *Clostridium* (>20% as maximum value, Tables S9 and S10). Regarding the order *Caryophanales* (former *Bacillales*) and the genus *Bacillus*, they were mostly identified in the sample KC10A (*S. stercoralis* negative), with a relative abundance of 2.38% and 2.14%, respectively (Tables S7 and S9). In fact, in all the other samples these taxa were found with a relative abundance lower than 1.5% (Tables S9 and S10), resulting in the least represented bacterial community. This fact could explain why the genus *Bacillus* and, consequently, the order *Caryophanales* were not identified in the samples using the approach based on the DADA2 pipeline (Tables S8 and S10). The general composition of the bacterial communities was not directly linked with *S. stercoralis* infection. However, all *S. stercoralis*-positive samples (KC4A, KC6A, and KC9A) showed very low percentages of the genus *Prevotella*, ranging between 0.17% and 1.06% for the data analyzed with the Kraken2/Bracken approach and between 0.21% and 1.31% according to the DADA2 analysis. In addition, one *S. stercoralis*-negative sample (KC1A) showed similarly low values for *Prevotella*; interestingly, this sample originated from a dog with frequent episodes of diarrhea (Table S2).

## 4. Discussion

The primary aim of this study was to assess a lab pipeline for the identification of *B. thuringensis* and its characterization for the presence of genes involved in the synthesis of Cry toxins, such as *cry5Ab*, *cry5Ac,* and *cry5Ba* genes [21]. While all six *B. cereus* controls were correctly identified, one out of the three *B. thuringiensis* positive controls (NRRL B-18765) was misidentified as *B. cereus* by MALDI-TOF, highlighting a possible discrepancy with the PCR/Sanger. This observation was deep-rooted extending the analysis to all the collection strains, and the *gyrB* genomic approach allowed differentiation between *B. cereus* and *B. thuringiensis*.

We then characterized all the strains by *ces*- and *cry5*-specific PCRs. As expected, only *B. cereus* had the genetic determinant for the emetic toxin cereulide (ces), whilst two positive *B. thuringiensis* controls (NRRL B-18765 and NRRL B-18400) confirmed all cry5 genes.

Of note, although *B. cereus* and *B. thuringiensis* have different pathogenicity and applications [30], still there are no available guidelines to reliably distinguish species and strains [3]. So far, different approaches such as protein crystallization, pulsed-field gel electrophoresis, and molecular typing were tested [3], but no reliable differentiation has been achieved with good results. A recent work assessed MALDI-TOF mass-spectrometry for differentiating closely related *Bacillus* species [31]. In this work, we chose *gyrB* as the target gene because it is also the target of the putative species-specific PCR developed by Yamada and colleagues (1999) [23] and we combined in-home PCR, Sanger sequencing, and MALDI-TOF approaches with the instruments and library currently used in our laboratory for a comprehensive identification of the strains. Our results highlight that the genomic approach (*gyrB* PCR and Sanger) is more accurate for *B. thuringiensis* identification compared to MALDI-TOF. Based on these results, we proceeded with our secondary objective and we used the approach for genomic characterization in order to investigate the potential presence of *B. thuringiensis* cry5 genes in dog stool. The data obtained from this analysis did not show a possible correlation between the presence of *S. stercoralis* infection and *B. thuringiensis*, as all samples from the dogs were negative for the toxin genes. To the best of our knowledge, to date, there is no evidence of in vivo correlation of *B. thuringiensis* with *S. stercoralis* or other parasites and only evidence of efficacy testing following treatment/administration [9,10]. Moreover, neither the general composition of the bacterial communities nor specific taxa (at the phylum, order, or genus level) were directly associated with *S. stercoralis* infection. In addition to *gyrB*- and *cry5*-specific PCRs, we used a *16S* metagenomics approach in order to detect the fecal bacteria composition. To reduce possible bias of biological/dietary/environmental interferents, we chose to analyze samples collected from dogs belonging to the same kennel, with sampling performed on the same day. However, being a small group, some differences in gender, age, physiological, and health status remain among the dogs leading to possible differences in fecal composition as well. By the way, these characteristics were not taken into account in this work. In order to strengthen the analysis, we conducted the bioinformatics examination using two different tools, in order to investigate how different approaches of read analysis can affect the interpretation of bacteria composition and community structure and try to evaluate the agreement between them. Kraken2|Brachen is based on alignment-free k-mer searches against a reference genome library [27], while DADA2 infers amplicon sequence variants (ASVs) and the identification at taxonomic level is based on sequence alignment against a reference database [29]. Overall, the two pipelines yielded almost identical compositions and relative frequencies at the phylum level for the samples analyzed, while some differences arose at the order and genus levels specifically for very-low-represented taxa, such as *Caryophanales* (former *Bacillales*) and *Bacillus*. The low sensitivity of DADA2 paired with SILVA for the characterization of samples at the genus level has been recently reported [32]. In addition, the authors confirmed the accuracy of the SILVA database for the investigation of the composition of microbial communities. Another aspect to consider is that the outcomes of DADA2 pipelines are directly dependent on the parameters chosen by the users for read filtering and trimming, which can affect the final identification of the taxa [33].

The two pipelines identified the presence of five different phyla among the samples analyzed, where the most abundant were *Bacteroidota* (former *Bacteroidetes* [34]) and *Bacillota* (former *Firmicutes* [34]). This is consistent with previous reports [35], which characterized the microbiota of 96 healthy dogs. *Bacteroidetes* and *Firmicutes*, together with *Fusobacterium*, represent the most important bacterial phyla in the gastrointestinal tract of dogs [36]. In particular, it is widely accepted that the *Firmicutes/Bacteroidetes* (F/B) ratio has an important role in maintaining intestinal homeostasis [37,38] and the evidence of this study is in accordance with that reported by Chaitman et al. (2020) [39], where the authors observed a significantly lower abundance of *Bacteroidetes* in dogs affected by acute diarrhea.

The overall composition of the fecal microbiome of the *Strongyloides*-infected and uninfected dogs enrolled in our study is in line with what is reported in the literature for dogs either under natural or experimental conditions [40,41]. In spite of the overall similarities in the composition of the fecal microbiota between *Strongyloides*-positive and -negative dogs, *Prevotella* was relatively mostly abundant in the uninfected compared to the infected group. There are no other data in the literature exploring the potential microbiota difference between *Strongyloides*-infected and uninfected dogs. Limited data have been described in chronic *S. stercoralis*-infected humans, showing significantly expanded populations of *Clostridia* and *Leuconostocaceae* [42] and an increasing presence of the *Ruminococcus* torques group [43]. On the other hand, discordant observations have been reported about *Prevotella* abundance in humans, with or without helminth infection [44]. Putatively the dogs enrolled in this study had *S. stercoralis* infection of a long duration due to re-infection from the environment, suggesting a potential correlation with *Prevotella* differential abundance between *Strongyloides*-infected and uninfected dogs.

Some limitations should be highlighted: (i) the small size of the dog cohort, which depended on the availability of samples in the context of our previous study [22] for which they were collected, and (ii) the uninfected dogs were not fully representative of the general canine population.

To conclude, here, we assessed an internal lab pipeline that aimed to characterize a list of *B. cereus* group species for three cry5 toxin genes and for the differential analysis of *B. thuringiensis* and *B. cereus*. Our results suggest that the genomic approach combining specific gyrB PCR and Sanger might be superior for *Bacillus* identification compared to the MALDI-TOF approach.

The specific PCRs and *16S* metagenomics analyses on dog stools showed no significant correlation between *B. thuringiensis* and the fecal microbiome, although a potential *Prevotella* differential abundance between *Strongyloides*-infected and uninfected dogs should be further explored. We provide preliminary descriptive results about fecal bacterial composition in dogs with and without *S. stercoralis* infection. Further investigations are needed in larger cohorts to investigate whether bacterial toxins might have a role in reducing environmental contamination by *S. stercoralis* larvae.

## Figures and Tables

**Table 1:** Results of *B. cereus* and *B. thuringiensis* identification with MALDI-TOF, PCR, and Sanger methods. *n* = 32 is the total of analyzed strains.

MALDI-TOF	PCR	Sanger	*n* (%)
*B. cereus*	*B. cereus*	*B. cereus*	21 (65.62)
*B. cereus*	*B. thuringiensis*	*B. thuringiensis*	7 (21.88)
*B. thuringiensis*	*B. thuringiensis*	*B. thuringiensis*	4 (12.50)

## Data Availability

All data generated or analyzed during this study are included in this published article (and its Supplementary Materials). The raw read sequences of the dog stool samples analyzed were deposited in the NCBI database under the BioProject ID PRJNA1134405 and the sequence of the gyrB gene obtained for the 44 *Bacillus* strains can be accessed in the NCBI database with the following accession number range: PQ014599–PQ014641.

## Supplementary Materials

The following supporting information can be downloaded at: https://www.mdpi.com/article/10.3390/microorganisms12081603/s1, Table S1. MALDI-TOF, PCR and Sanger sequencing results for *B. cereus* group strains; Table S2. Dataset of the study dogs; Table S3. PCR results in stool samples; Table S4. Number of reads analyzed for each stool sample by 16S metagenomics approach; Table S5. Kraken2/Bracken—Silva results at phylum level in the fecal samples of 10 dogs; Table S6. DADA2-Silva results at phylum level in the fecal samples of 10 dogs; Table S7. Kraken2/Bracken -Silva results at order level in the fecal samples of 10 dogs; Table S8. DADA2-Silva results at order level in the fecal samples of 10 dogs; Table S9. Kraken2/Bracken -Silva results at genus level in the fecal samples of 10 dogs; Table S10. DADA2-Silva results at genus level in the fecal samples of 10 dogs.
