# Supplementary material for: A Pilot Study for the Characterization of Bacillus spp. and Analysis of Possible B. thuringiensis/Strongyloides stercoralis Correlation
Source: Microorganisms. 2024 Aug 6;12(8):1603. doi: 10.3390/microorganisms12081603 (PMC11356623; doi:10.3390/microorganisms12081603)
Supplement: Supplementary file 1 [file microorganisms-12-01603-s001.zip › microorganisms-3126191-supplementary/Supplementary material legend.pdf]

Supplementary material legend

Table S1. MALDI-TOF, PCR and Sanger sequencing results for *B. cereus* group strains.

Table S2. Dataset of the study dogs.

Table S3. PCR results in stool samples

Table S4. Number of reads analysed for each stool sample by 16S metagenomics approach.

Table S5. Kraken2|Bracken -silva results at phylum level in the fecal samples of 10 dogs.

Table S6. DADA2-silva results at phylum level in the fecal samples of 10 dogs.

Table S7. Kraken2|Bracken -silva results at order level in the fecal samples of 10 dogs.

Table S8. DADA2-silva results at order level in the fecal samples of 10 dogs.

Table S9. Kraken2|Bracken -silva results at genus level in the fecal samples of 10 dogs.

Table S10. DADA2-silva results at genus level in the fecal samples of 10 dogs.
